# Identification of 2-(4-*N,N*-Dimethylaminophenyl)-5-methyl-1-phenethyl-1*H*-benzimidazole targeting HIV-1 CA capsid protein and inhibiting HIV-1 replication *in cellulo*

**DOI:** 10.1186/s40360-022-00581-7

**Published:** 2022-06-28

**Authors:** Guzmán Alvarez, Lisa van Pul, Xavier Robert, Zoraima Artía, Ad C. van Nuenen, Mathieu Long, Natalia Sierra, Williams Porcal, Neeltje A. Kootstra, Christophe Guillon

**Affiliations:** 1 Laboratorio de Moléculas Bioactivas, Departamento de Ciencias Biológicas, CENUR Litoral Norte, Universidad de La República, 60000 Paysandú, Uruguay; 2 Department of Experimental Immunology, Amsterdam UMC, Amsterdam Infection & Immunity Institute, University of Amsterdam, 1105 AZ Amsterdam, the Netherlands; 3 Retroviruses and Structural Biochemistry, UMR5086, Université de Lyon, CNRS, MMSB, 69367 Lyon, France; 4 Departamento de Química Orgánica, Facultad de Química, Universidad de La República, 11800 Montevideo, Uruguay

**Keywords:** HIV-1, Capsid, CA, p24, Inhibitor, Benzimidazole

## Abstract

The capsid (CA) subunit of the HIV-1 Gag polyprotein is involved in several steps of the viral cycle, from the assembly of new viral particles to the protection of the viral genome until it enters into the nucleus of newly infected cells. As such, it represents an interesting therapeutic target to tackle HIV infection. In this study, we screened hundreds of compounds with a low cost of synthesis for their ability to interfere with Gag assembly in vitro. Representatives of the most promising families of compounds were then tested for their ability to inhibit HIV-1 replication in cellulo. From these molecules, a hit compound from the benzimidazole family with high metabolic stability and low toxicity, 2-(4-N,N-dimethylaminophenyl)-5-methyl-1-phenethyl-1H-benzimidazole (696), appeared to block HIV-1 replication with an IC50 of 3 µM. Quantitative PCR experiments demonstrated that 696 does not block HIV-1 infection before the end of reverse transcription, and molecular docking confirmed that 696 is likely to bind at the interface between two monomers of CA and interfere with capsid oligomerization. Altogether, 696 represents a promising lead molecule for the development of a new series of HIV-1 inhibitors.

**Supplementary Information:**

The online version contains supplementary material available at 10.1186/s40360-022-00581-7.

## Background

Human Immunodeficiency Virus type 1 (HIV-1), the causative agent of AIDS, is a major concern of public health with more than 37 million infected people worldwide [1]. In front of this pandemic, numerous treatments have been developed, against which HIV-1 is prone to resistance development given its intrinsic variability [2]. Thus, new viral therapeutic targets with limited variability are sought to try to circumvent this problem. One of the most conserved regions of HIV-1 sequence is the Gag polyprotein [3]. Gag is a structural protein that is responsible for the architecture of the viral particle, the protection of the viral genome from cellular nucleases, and the import of the reverse-transcribed DNA into the nucleus before its integration in the genome of the infected cells. In particular, in the late phase of viral replication, the capsid protein (CA) of the Gag polyprotein is oligomerizing into pentamers and hexamers to the mature viral core of the virion which stability is necessary for effective infection [4]. Moreover, CA is interacting with several cellular factors to play different roles during the early phase of the viral replication cycle, from uncoating to trafficking and entering the nucleus of the infected cells [5]. Among others, HIV-1 CA interacts with Cyclophilin A, Nucleoporins 153 and 358, and polyadenylation specificity factor 6 (CPSF6)[6–10]. It can also interact with restriction factor Trim5α to allow the evasion of HIV from immune innate mechanisms [11]. The fact that CA is important for both early and late phases of the infection makes it a target of choice to develop anti-HIV compounds [12]. Moreover, these pleiotropic roles impose a strong selection to maintain all these functions, resulting in low sequence diversity within a single retroviral species and a conserved typical α-helical structure between retroviruses [13–15]. It is composed of two domains: the N-terminal domain (NTD) is made up of 7 α-helices while the C-terminal domain (CTD) has 4 α-helices. FIV (feline immunodeficiency virus) and HIV-1 CA also possess an N-terminal β-hairpin which seems to be involved in the entry of nucleotides for the reverse transcription of the viral RNA genome into double-stranded DNA [13, 16]. NTD and CTD of CA are separated by a flexible linker which allows the two domains to undergo relative spatial rearrangements during CA oligomerization. HIV-1 CA interactions with adjacent monomers to form the pentamers and hexamers involve both NTD-NTD, CTD-CTD, and NTD-CTD interactions, and CA-CA interactions are also needed to assemble the oligomers and form the fullerene-shaped mature viral core [4, 17–19].

Therefore, the emergence of viruses with escape mutations to anti-CA treatments could be hindered by these sequence and structural constraints, reinforcing the interest of CA as a therapeutic target for HIV infection. Peptides, peptidomimetics, or small molecules targeting CA have been developed which could inhibit HIV in vitro and are mostly binding three regions of CA [20]: hydrophobic pockets in the NTD, a groove in the CTD, or a pocket formed between the NTD of one monomer and the CTD of an adjacent monomer in a CA hexamer [21–25]. These various targeted regions are reflecting multiple mechanisms of inhibition, from blocking of CA-CA interactions during virus assembly to interference with post-entry events through the stabilization of the viral core or competition with cellular partners [20, 22]. Although a recent paper has demonstrated the clinical potential of CA as a therapeutic target [26], no CA inhibitor is available yet on the market to tackle HIV infection. The low metabolic stability of some compounds [22] and high costs of production are among the main obstacles to their development for clinical use. Thus, the quest for new compounds against HIV-1 CA is still ongoing.

More than 400 compounds from our chemical library described elsewhere have been tested for their ability to interfere with CA assembly. Then from the data previously acquired from those 400 molecules tested in CA from FIV, we selected 10 compounds for their structural relationship with known CA HIV-1 inhibitors [27–31]. From those molecules, 5 compounds demonstrated interference with the assembly of HIV-1 *CA *in vitro One hit molecule, compound 696, which was recently shown by NMR to bind FIV capsid protein [27], demonstrated an inhibitory effect on HIV-1 replication in cellulo, with an IC_50_ of 3 µM. Docking experiments with this hit compound demonstrated its binding to the NTD of HIV-1 CA, in the zone of interaction of CA with its cellular partner Nup153 which is also targeted by the structurally-related inhibitor PF74 [21]. A comparison of the binding site of the PF74 and the possible interaction region of 696 suggests a different inhibition mechanism between PF74 and our compound 696. Also, PF74 accelerated the oligomerization in vitro, and 696 inhibited these. Moreover, 696 shows high metabolic stability and low toxicity, which makes it an interesting lead molecule to optimize for the development of a new class of low-cost HIV CA inhibitors.

## Results and discussion

### Screening of the chemical library for CA assembly inhibition in vitro

**Fig. 1:**
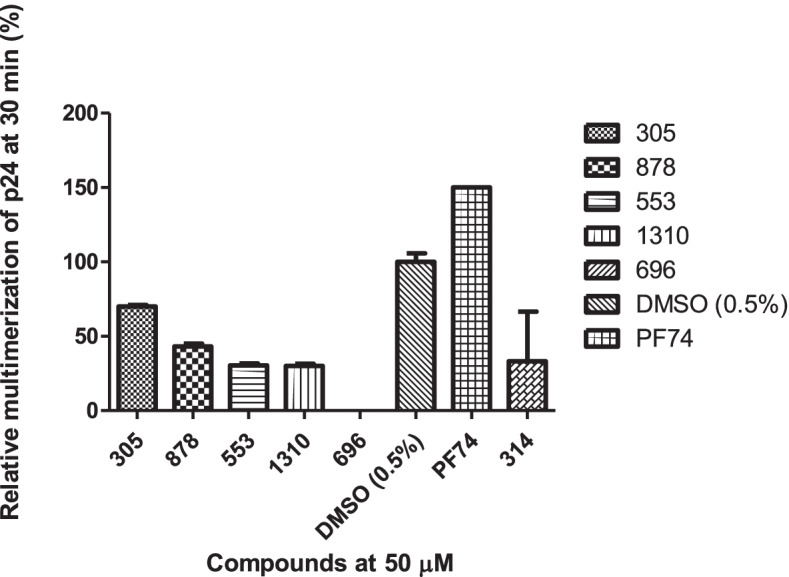
Inhibition of HIV-1 CA assembly in vitro at 50 µM. Results are expressed as the percentage of assembly at 30 min of measure, compared to the maximum assembly-level reached with untreated CA(negative control DMSO 0.5%). Data for PF74 was acquired in the same condition from Xu J. P. 2018 et al. [31]

**Fig. 2:**
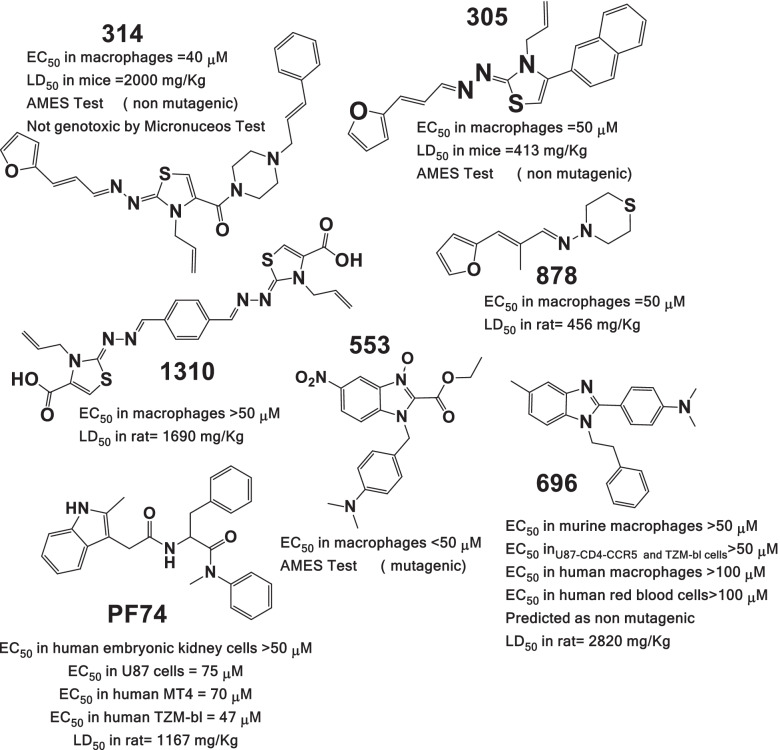
Chemical structures of the best compounds and toxicology summary [29, 31–33] compared to reference anti-CA drug PF-74. Detailed synthesis and characterization of 696 and 314 are available in supporting information. EC_50_ (concentration of a drug that gives half-maximal response) corresponds to the cytotoxicity of the compounds. LD_50_ correspond to the oral acute toxicity in rat or mice, and AMES test predicts mutagenicity

More than 400 compounds from our chemical library described elsewhere [28–30] have been tested for their ability to interfere with CA assembly. As the assembly inhibition assay requires high protein and molecule concentrations, with the concomitant solubility problems, a dose–response curve could not be determined for this assay as the 1:1 protein: compound ratio is unreachable. However, it is a common first-line screening strategy for anti-assembly molecules to select molecules to be tested *in cellulo* and/or in vivo [26]. Then from the data previously acquired from those 400 molecules tested in CA from FIV, we selected 10 compounds for their structural relationship with known CA HIV-1 inhibitors [30]. From those molecules, 5 compounds demonstrated interference with the assembly of HIV-1 *CA *in vitro (Fig. 1), namely compounds 305, 553, 878, 696, and 1310 (Fig. 2).

Compound 314 showed an inconsistent behavior because of its insolubility at concentrations higher than 10 µM. Compounds 314 and 305 are thiazolidene hydrazines designed as antiparasitic compounds targeting *Trypanosoma cruzi* (Fig. 2). Compounds 1310 and 878 are derivatives from the same rational design as compound 314, but these were not active against *T. cruzi* and have no recognized biological activity [32]. Notably, compounds 696 and 553 are structurally related to CA inhibitor PF74. However, 553 had high unspecific cytotoxicity in murine macrophages 100% at 50 µM in 24 h. Also, it has a nitro group which is generally associated with toxicity and interference with liver metabolism and was not considered for *in cellulo* experiments, also it was tested positive in the Ames test in silico (Fig. 2).

### Toxicology profile of the active compounds

The cytotoxicity of the compounds was assayed in different types of cells (TZM-bl cells, U87-CD4-CCR5, murine macrophages (J774.1), and THP-1 monocytes) at 25, 50, and 100 µM (Fig. 2). All of the compounds demonstrated EC_50_ of more than 50 µM, except for compound 553. Compound 314 has the most complete toxicology profile because was preclinically developed for Chagas disease infection, as an antiparasitic drug. This one also demonstrates good toxicology profiles, with no mutagenicity and no genotoxicity, also low oral toxicity in vivo. Compound 696 also demonstrates low unspecific toxicity. It has lower oral toxicity than PF74. Was predicted as no mutagenic by the TEST software.

### Inhibitory activity of selected compounds against HIV-1 replication in cellulo

**Fig. 3:**
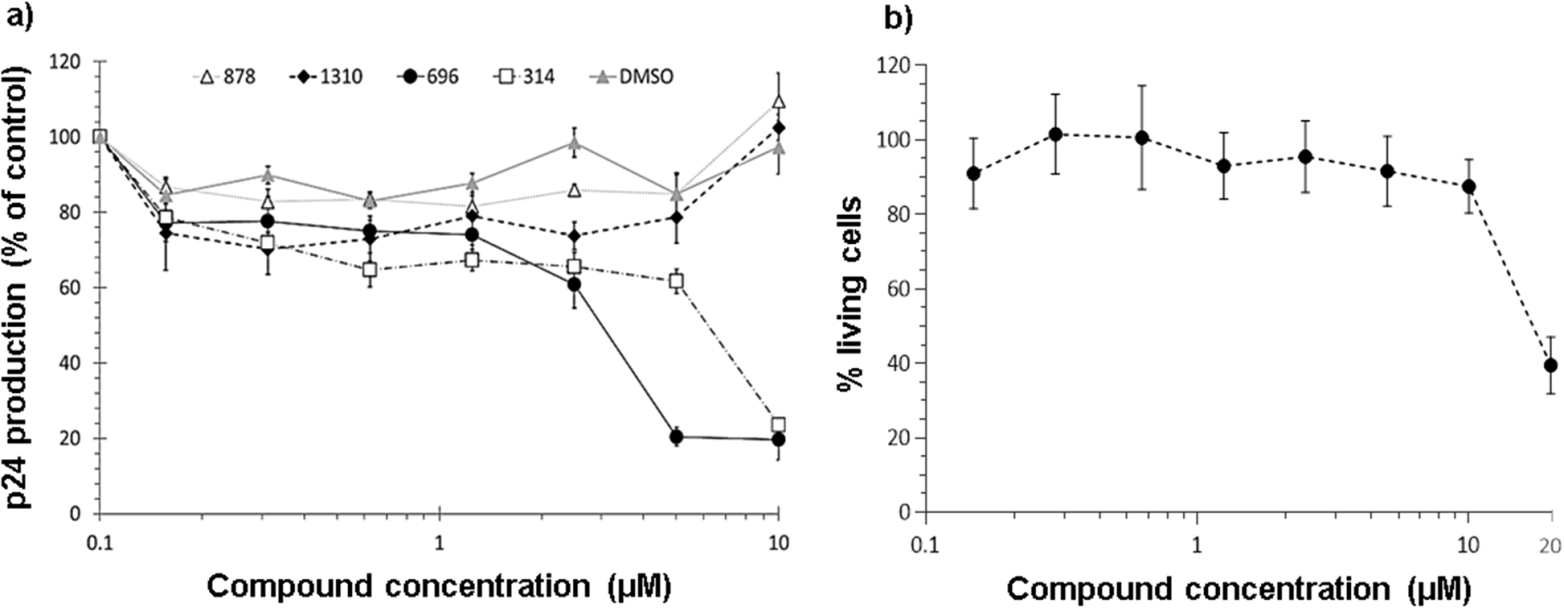
**a** Dose–response inhibition of HIV-1 replication in U87-CD4-CCR5 cells after 11 days. Each curve displays the mean and SD of 4 independent wells of a representative experiment (*n* = 3). Viral replication was determined by p24 production in the culture supernatant and expressed as % of the DMSO-treated control. **b** Toxicity of compound 696 on U87-CD4-CCR5 cells measured by MTT assay after 11 days displayed as percentage live cells (mean and SD of 4 wells) relative to control

Based on the compounds which showed an inhibitory effect in our assembly assay (Fig. 1), we selected molecules from our chemical library related to these lead compounds, favoring those with good solubility and low toxicity. This led us to consider 22 molecules (Table S2) in total that were tested *in cellulo* for their ability to inhibit HIV-1 replication. Among the 22 compounds, only two compounds showed a clear dose–response inhibition of HIV-1 replication, compounds 314 and 696. Compound 696 showed inhibition of HIV-1 replication in U87-CD4-CCR5 cells of more than 80% at the two highest concentrations tested after 11 days (Fig. 3a). As demonstrated using MTT assay, this effect was not due to the possible toxicity of compound 696 in the range of concentration we used (Fig. 3b). Thus, with an IC_50_ of 2.3 ± 0.6 µM on day 11, 696 demonstrates a selective activity of at least 10 times between mammalian cells (EC_50_ > 50 µM, Fig. 2) and antiviral activity (EC_50_/IC_50_ > 21). Also compound 696 had low toxicity on murine macrophages EC_50_ > 50 µM (at 48 hs), EC_50_ inU87-CD4-CCR5 (11 days) and TZM-bl cells > 50 mM (48hs), EC50 in human macrophages > 100 mM (48hs), EC_50_ in human red blood cells > 100 mM (24hs).

Among the other compounds, 314 showed inhibition only at 10 µM, which was not sufficient for the calculation of an IC_50_. A trend towards inhibition of HIV-1 replication at low concentrations for compounds 878 and 1310 was observed. The results obtained with 878 and 1310 could not be confirmed at high concentrations because of solubility problems. In particular, compound 1310 has two ionizable groups, which may interfere with its permeability into the cells. It will be interesting to consider these two compounds for future chemical modifications, such as esterification with methanol of compound 1310 or a different solubilization approach with compound 878. The remaining compounds tested showed no inhibition of HIV-1 replication in U87-CD4-CCR5 cells (data not shown). No p24 production was detected in the AZT (10 µM) control (data not shown).

**Fig. 4:**
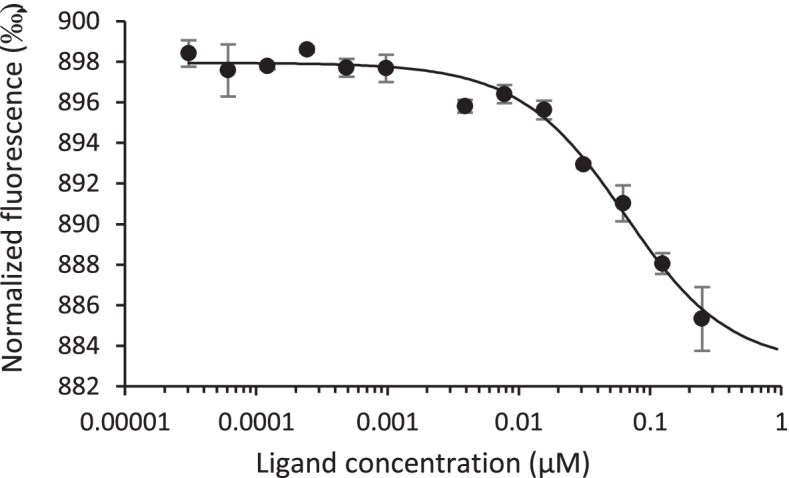
Measure of the affinity for HIV-1 CA of 696 by MST. It is showed one representative experiment out of three

We have recently demonstrated the binding and inhibitory effect of 696 on FIV capsid assembly in vitro [27]. Thus, we investigated the interaction of 696 with HIV-1 CA by microscale thermophoresis (MST). Interestingly, specific binding of compound 696 to monomeric HIV-1 CA was detectable with an estimated K_D_ of 69 ± 6 µM (Fig. 4). On the opposite, although showing some inhibitory effects at high concentrations, compound 314 displayed no specific binding for HIV-1 CA at 200 µM (data not shown). This suggests that the effect of 314 on HIV-1 replication might not be specific to CA inhibition and that this compound might interfere with another step of viral replication. As it is an efficient inhibitor of some parasite proteases [33], an inhibitory effect of 314 against HIV-1 protease will be worth exploring. Thus, we focused on compound 696 as it showed a clear dose–response inhibition of HIV-1 replication *in cellulo* and a specific affinity for HIV-1 CA in the micromolar range.

### Compound 696 does not block HIV-1 infection before the end of the reverse transcription

Various mechanisms of action have been described so far for CA inhibitors (reviewed in [20]): inhibition of the formation of the viral core in the late steps of viral replication by inhibition of CA/CA interactions will lead to the production of non-infectious viral particles, while inhibition of early stages of infection, during uncoating and/or nuclear import of the capsid will lead to abortive infection before integration and will block further viral replication.

**Fig. 5:**
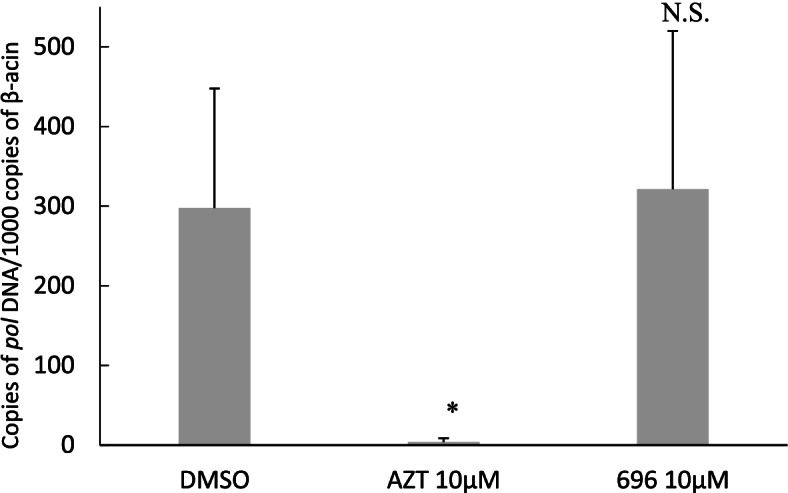
Inhibition by 696 of early steps in the viral replication cycle by the analysis of reverse transcription by qPCR detecting viral DNA. Each bar displays the mean and SD of 4 wells of qPCR for the HIV-1 pol gene of a representative two independent experiments (see Materials and methods). Stars demonstrate the significance of the difference compared to DMSO-treated samples (unpaired two tails Mann–Whitney test) with *: *p* < 0.05 and N.S.: non-significant (*p* > 0.05). Results are expressed as the copy number of pol viral DNA per 1000 copies of β-actin

As inhibition of infection of U87-CD4-CCR5 cells can reflect inhibition at both early and late steps of infection, we evaluated specifically the effect of compound 696 on the early phase of the viral cycle. To check if the addition of the CA inhibitor inhibited HIV-1 reverse transcription, qPCR was performed on cells preincubated with 696 for 18 h (Fig. 5) before infection.

The nucleoside analog Zidovudine (AZT) was used as a positive control for inhibition of reverse transcription. Pre-incubation of the cells with AZT resulted in an efficient inhibition of HIV-1 reverse transcription (*p* = 0.029), while compound 696 did not show any significant effect. Preincubation of the cells with the compound for 18 h did not show any significant effect of 696 either (data not shown). This suggests that 696 does not inhibit viral replication during the steps of viral replication which are taking place between entry and reverse transcription.

### Modelling the mechanisms of inhibition by 696

**Fig. 6:**
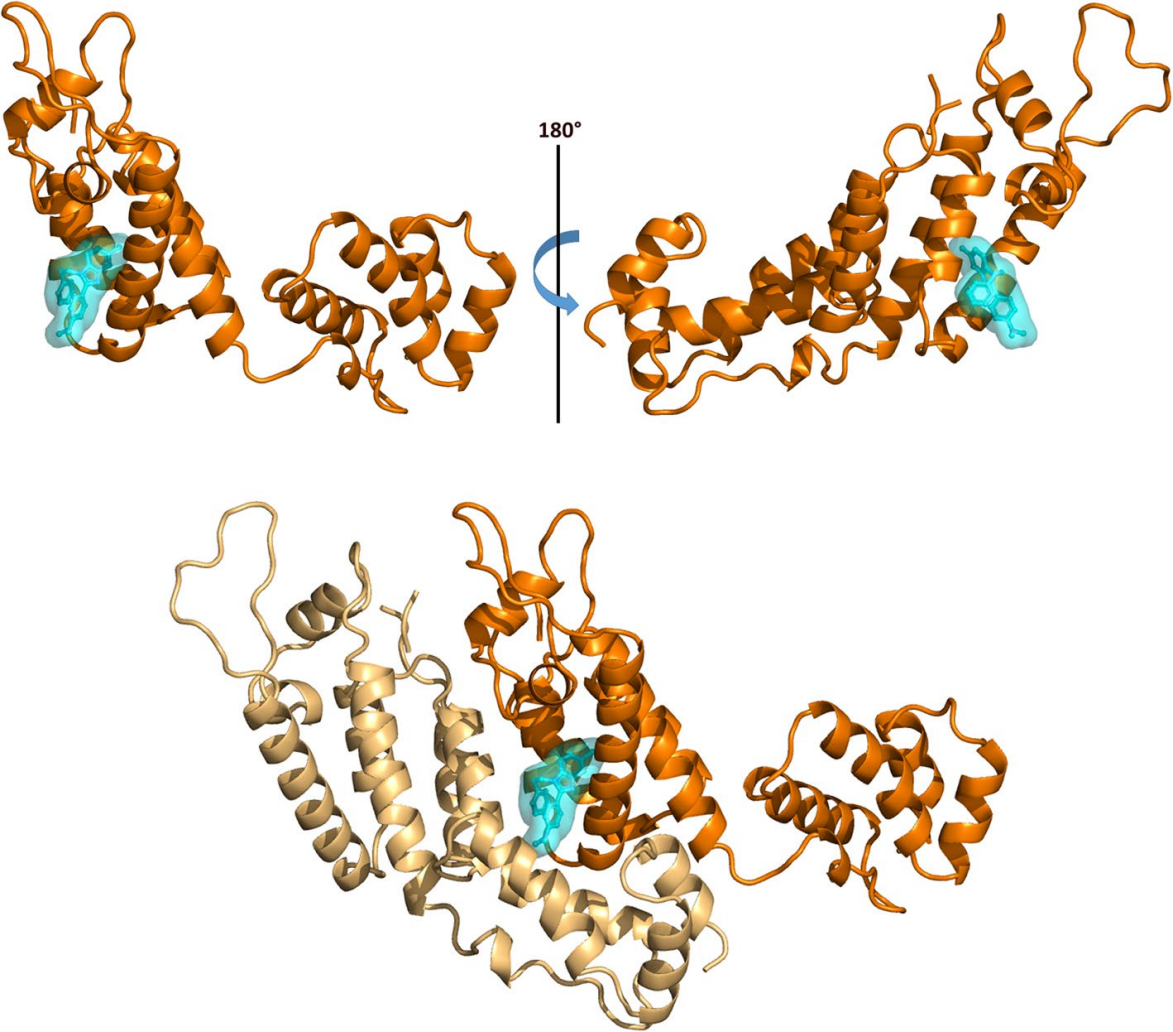
Molecular docking of compound 696 on HIV-1 CA monomer (top) or dimer (bottom). Compound 696 and its surfaces are displayed in cyan and the chain of the dimer used for docking is displayed in dark orange. HIV-1 CA monomers and dimers are extracted from PDB entry 4XFZ [19]

**Fig. 7:**
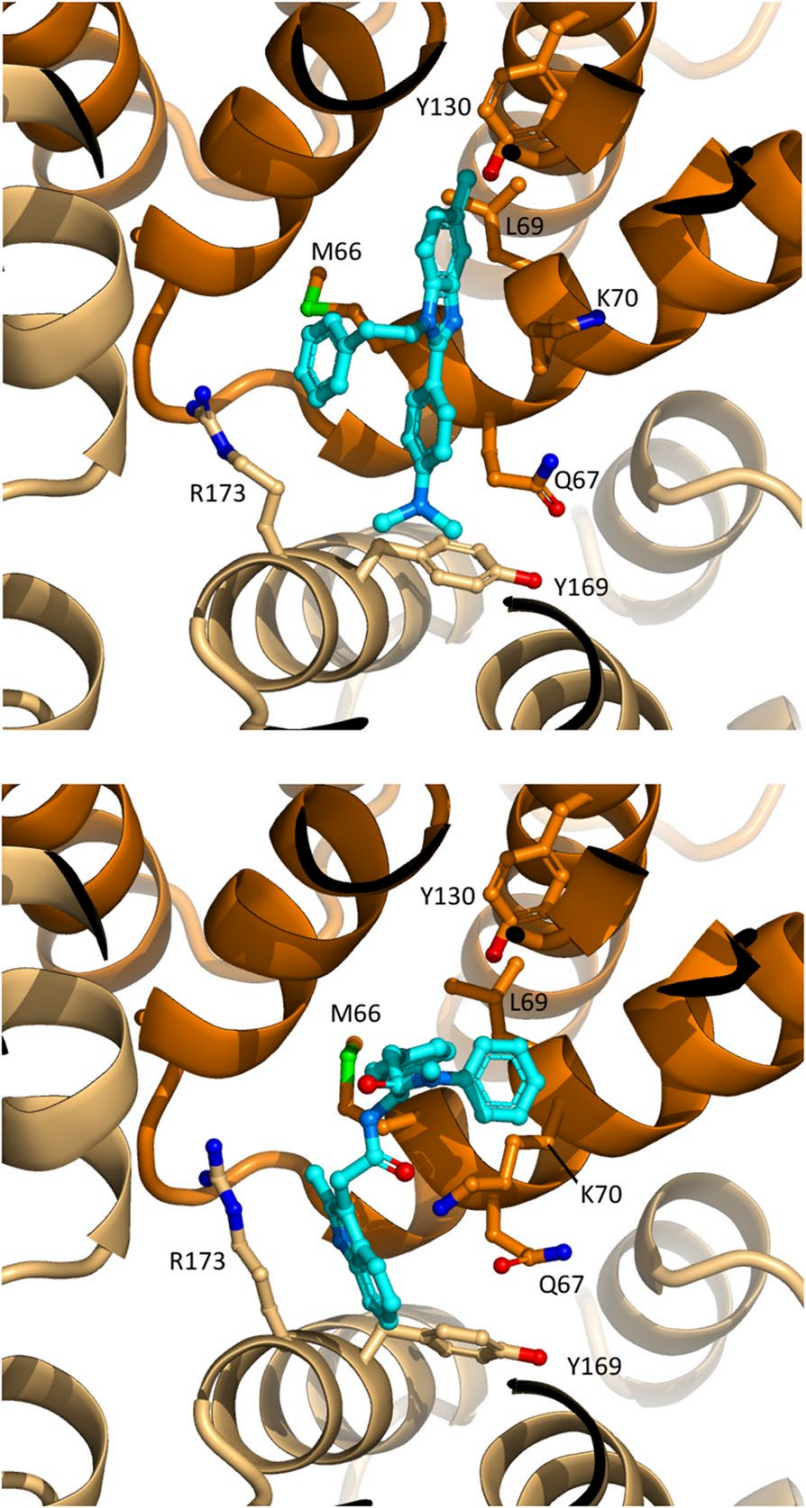
Molecular docking of compound 696 in a CA dimeric interface (top) compared to the published structure of PF74 in a complex with a CA hexamer (bottom, PDB entry 4XFZ [19]). The coloring of the amino-acid chains is identical to Fig. 6

To understand the molecular mechanisms of inhibition, compound 696 was computationally docked against a monomer of HIV-1 CA (Fig. 6). The molecular docking for 696 suggests that this compound binds to the NTD of CA, in a zone that is involved in protein: protein interactions during HIV-1 CA oligomerization [19]. Thus, we positioned compound 696 at the interface between two monomers of an HIV-1 CA hexamer. Compound 696 appears to bind at the interface between monomers (Fig. 7). This suggests that this compound may inhibit HIV-1 replication in cells by interfering with Gag:Gag interactions and/or capsid transportation during the viral cycle.

Noteworthy, PF74 and 696 are structurally related and PF74 has been shown to inhibit HIV-1 by targeting the same area in a rather similar orientation (Fig. 7). The main difference between the two compounds is the extrusion of PF74, which presents a larger interface of interaction with the second monomer of CA (130 Ǻ2) than 696 (16 Ǻ2), as calculated by PISA [35].

Interestingly, this zone is the binding site of cellular partners Nup153 and CPSF6, which are involved in the import of the CA or its content from the cytoplasm to the nucleus before HIV-1 integration [8–10].

Indeed, PF74 has been described as inhibiting the uncoating step by stabilizing the assembled viral capsid, thereby targeting early steps of viral replication and resulting in the blockade of HIV-1 integration [21, 36]. Our molecular docking strategy, which proved successful in identifying the binding site of 696 of FIV CA [27], suggests that 696 is binding at the same site as PF74 on HIV-1 CA However, we showed that this compound does not inhibit the early steps of HIV-1 replication, before reverse transcription (Fig. 5). This could come from a weaker interaction of 696 with HIV-1 CA, suggested by the smaller surface of interaction with the second monomer of CA compared to PF74 (Fig. 7). It could also reflect that 696, as PF74, could interfere with the import of the HIV-1 capsid in the nucleus at the pre-integration step [34]. Structure-based optimization of 696 to increase this interaction and more detailed experiments to address which step of the HIV-1 viral cycle is inhibited, either pre-integration events and/or assembly itself, will be sought to clarify this point.

Noteworthy, PF74 has been described as having low metabolic stability [22] (Table 1), which is not the case here as 696 was stable for 4 h under microsomal and cytosolic fraction derived from rat liver, as checked by Thin Layer Chromatography by the absence of new metabolites (Figure S2 in supporting material). 696 demonstrated also a selective activity of at least 10 times between mammalian cell and antiviral activity (Figs. 2 and 3). These observations, together with the low toxicity effects predicted in vivo (see supporting information) make this compound a safe hit candidate for a drug development process. Moreover, 696 is a benzimidazole derivative, which is encouraging as such derivatives are among the most frequently used ring systems in drugs listed by the US FDA [37]. Furthermore, 696 has the advantages of easy and low-cost preparation (see supporting information). This is a crucial point, as the affordability of prescription drugs is a limitation in low-income countries with high HIV-1 prevalence, which host most of the HIV-infected individuals worldwide. Nowadays, that is the real limitation in HIV control because most people cannot afford the costs of the actual treatments. This needs to be kept in mind for the upcoming optimization based on compound 696 as a hit molecule, before being used as an anti-HIV-1 therapeutic molecule.

**Table 1:**
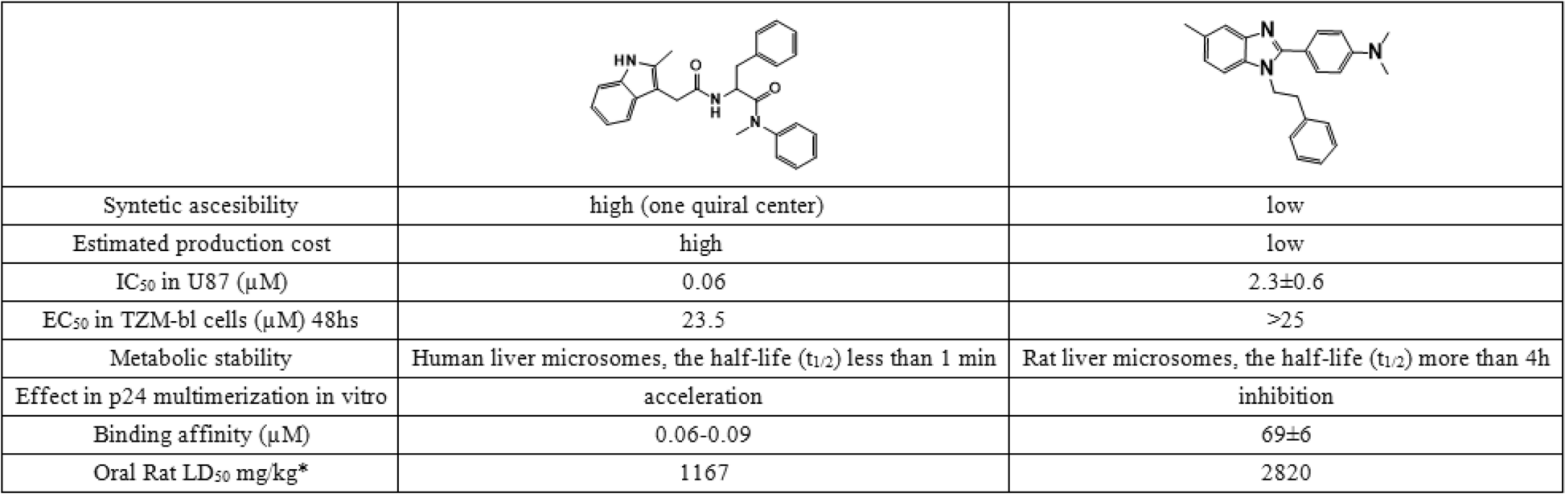
Comparison pharmacology profiles between PF74 and 696

*Calculated by Toxicity Estimation Software Tool (TEST)

## Materials and methods

### Description of the compounds

The herein studied compounds were selected using the following criteria: i) agents inhibiting CA assembly of FIV in vitro, ii) compounds with strong activity in other pathogens with low-cost production, or iii) compounds with low unspecific toxicity. Then from the chemical collection with around 2000 exclusive compounds designed by our team, we selected 10 compounds belonging to diverse structural families: steroids, quinoxalines, curcuminoids, thiazolidene hydrazines, and benzimidazole. Then, based on the compound which showed an inhibitory effect in our assembly assay, we extended our selection with additional molecules related to these lead compounds to finally consider 22 molecules in total for their inhibitory effect on HIV-1 replication in U87 cells. The chemical synthesis of these molecules followed the previously reported procedures [28, 30, 33, 38, 39]. Synthesis and in-depth characterization of the two compounds 696 [27] and 314, which are thoroughly analyzed in this study, are available as supporting information.

### Liver fraction stability studies

For the determination of liver fraction stability, rat liver microsomal and cytosolic proteins were used (MOLTOX™ manufactures products, North Carolina, US). Rat liver microsomal and cytosolic proteins were used according to the manufacturer’s recommendations and the methodology previously described [40]. The protein content of the microsomal and cytosolic fractions was determined by the bicinchoninic acid assay from Sigma (Schnelldorf, Germany), as suggested by the manufacturer. The final concentration of 696 in the aqueous medium was 100 µM and was prepared from a stock solution in DMSO. The solutions were further homogenized and incubated at 37 ^o^ C for 1 to 4 h following the OECD recommendation for drug development. After that, thin-layer chromatography of ethyl acetate extracts was performed to evaluate the presence of metabolites.

### Calculation of the pharmacokinetic parameters [40, 41]

The predictions were performed as described before [42] with the open-access SwissADME software (http://www.swissadme.ch, accessed on 25 February 2020), a tool that allows the prediction of different pharmacokinetic parameters such as water solubility, lipophilicity, gastrointestinal absorption, bioavailability, etc. (see supporting information). The oral median lethal dose (LD_50_) in rats was estimated using the Toxicity Estimation Software Tool (TEST). For both of them, the SMILES codes, generated with the ChemBioOffice 2010 program, were used.

### Red blood cell lysis assay [32]

Human blood (blood discarded from the blood bank) collected in sodium citrate solution (3.8%) was centrifuged at 200 × g for 10 min at 4 °C. The plasma supernatant was removed and erythrocytes were suspended in ice-cold PBS. The cells were again centrifuged at 200 × g for 10 min at 4 °C. This procedure was repeated two more times to ensure the removal of any released hemoglobin. Once the supernatant was removed after the last wash, the cells were suspended in PBS to 2% w/v red blood cell solution. A volume of 400 μL of compound 696 to be analyzed, in PBS (final doses 50, 100, and 200 μM), negative control (solution of PBS), or Amphotericin B (AmpB) (final dose 1.5 μM), was added to the 2% w/v red blood cell solution. Ten replicates for each concentration were done and incubated for 24 h at 37 °C before analysis. Complete hemolysis was attained using neat water yielding the 100% control value (positive control). The release of hemoglobin into the supernatant was determined spectrophotometrically at 405 nm using a multiplate reader VarioskanTM Flash Multimode Reader (Thermo Scientific, MA, US). Results are expressed as the percentage of total hemoglobin released in the presence of the compounds. This percentage was calculated using the equation percentage hemolysis (%) = [(A_1_ − A_0_)/(A_w_)] × 100, where A_1_ is the absorbance at 405 nm of the test sample at t = 24 h, A_0_ is the absorbance at 405 nm of the test sample at t = 0 h, and A_w_ is the absorbance at 405 nm of the positive control (water) at t = 24 h. The EC_50_ was defined as the drug concentration at which 50% of the cells were not lysed relative to the control (solution of PBS) and was determined by analysis using OriginLab8.5® sigmoidal regression (% of not lysed cells compared to the logarithm of the compound concentration). The experiments were done in quintuplicate. AmpB was used as a positive control.

### Cytotoxicity in mammalian cells

TZM-bl cells, U87-CD4-CCR5, murine macrophages (J774.1), and THP-1 monocytes (ATCC®TIB-202™) were grown following ATCC recommendations. Cells were seeded in a 96-well plate (10^4^ cells in 200 µL culture medium) and incubated at 37^o^ C in a 5% CO_2_ atmosphere for 48 h, to allow cell adhesion before drug testing. For stimulation (THP-1 monocytes), cells were incubated with 100 nM of phorbol 12-myristate 13-acetate (PMA) for 48 h. PMA was then washed and cells were left for an additional 24 h, 48 h or11 days (only U87 cells) with growth media containing compound 696 at 10, 25, 50, and 100 µM. A vehicle for control (0.4% DMSO) and negative wells (cells in medium) was used in each test. Cell viability was then assessed by measuring the mitochondria-dependent reduction of MTT (3-(4,5-dimethylthiazol-2-yl)-2,5-diphenyltetrazolium bromide) to formazan. For this purpose, MTT in sterile PBS (0.2% glucose), pH 7.4, was added to the macrophages to achieve a final concentration of 0.1 mg/mL, and the cells were incubated at 37 ^o^ C for 3 h. After removing the medium, formazan crystals were dissolved in 180 µL of DMSO and 20µL of MTT buffer (0.1 M glycine, 0.1 M NaCl, 0.5 mM EDTA, pH 10.5), and the absorbance at 560 nm was measured. The EC_50_ was defined as the drug concentration at which 50% of the cells were viable relative to the control (0.4% DMSO), and was determined by analysis using OriginLab8.5® sigmoidal regression (% of viable cells compared to the logarithm of the compound concentration)[39]. Tests were performed in triplicate, in two independent experiments.

### Expression and purification of HIV-1 CA

The HIV-1 CA protein was expressed and purified as described for FIV CA with adaptations [31, 43]. Escherichia coli cells (BL2I (DE3) pLysS, Lucigen, WI, US) transformed with HIV-1 pNL4-3 CA expressing plasmid (cordially provided by Professor Michael Summers, University of Maryland, US) were grown in Terrific Broth Medium (Sigma-Aldrich, Schnelldorf, Germany), supplemented with 50 mg/mL of ampicillin, at 37 °C. When cells reached an OD value between 0.3 and 0.4 at 600 nm, the expression of CA was induced by the addition of 1 mM isopropyl-D-1-thiogalactopyranoside (IPTG, Euromedex, Souffelweyersheim, France) for 20 h at 25 °C, then cells were harvested by centrifugation (7000 × g) and the pellets were stored overnight at -20 °C. The pellet was then treated with lysis buffer (50 mM NaH_2_PO_4_, 300 mM NaCl, pH 8.5, lysozyme 1 mg/mL, DNase I 1 mg/mL, protease inhibitor cocktail 80 mg/mL, MgCl_2_ 4 mM) for 1 h at 0 °C, followed by 4 steps of sonication (1 min then 30 s of rest) at full power (Omni Sonic Ruptor 400 ultrasonic Homogenizer, PerkinElmer, MA, US). The clarification step was performed twice by centrifugation at 10,000 × g for 20 min and the supernatant was filtered on a 0.45 µm filter. The purification of HIV-1 CA protein was performed by nickel affinity chromatography using batch incubation with Ni^2+^TED resin (0.15 g of resin per g of pellet, Macherey–Nagel, Düren, Germany), then loaded onto a gravity column. The column was washed three times with LEW buffer (50 mM NaH_2_PO4, 300 mM NaCl, pH 8.5), and the elution was performed with LEW buffer containing increasing concentrations of imidazole (5 mM, 50 mM, 500 mM). The concentration of CA was quantified by spectrophotometry at 280 nm using a Nanodrop (Thermo Fisher, MA, US). The purity of the protein at homogeneity was checked using SDS-PAGE analysis before further processing. Buffer exchange, using ultrafiltration devices (10 kD MWCO, Sartorius, Gotinga, Germany), was performed against a Phosphate/NaCl Buffer (NaH_2_PO_4_/Na_2_HPO_4_ 50 mM, NaCl 100 mM, pH 7.4).

### Assembly inhibition assay

The CA assembly assay was performed in a final volume of 70 µL in 384-well plates, with a final concentration of 5 mg/mL of purified recombinant HIV-1 CA in Phosphate/NaCl Buffer (NaH_2_PO_4_/Na_2_HPO_4_ 50 mM, NaCl 100 mM, pH 7.4), as described for FIV CA [27, 31]. Absorbance was measured at 340 nm every 30 s in a multiplate reader VarioskanTM Flash Multimode Reader (Thermo Scientific, MA, US) at 37 °C for 30 min. The compounds were tested at a fixed initial dose of 25 or 50 µM (at 0.5% DMSO [v/v]) by adding 1 µL of the concentrated ligand to the well before starting the measurement. The controls were CA at 5 mg/mL in only 0.5% DMSO [v/v] and CA without DMSO. The criteria to select active compounds were the observation of at least 50% of assembly inhibition at these doses, based on the reduction of the OD value at 340 nm at the plateau value compared to the controls.

### Cell lines

U87-CD4-CCR5 cells [44] and TZM-bl cells [45] were obtained through the NIH AIDS Reagent Program Division of AIDS, NIAID, NIH, and were cultured in Iscove’s Modified Dulbecco Medium (IMDM) (Lonza, Basel, Switzerland) supplemented with 10% [v/v] inactivated fetal calf serum (Hyclone- Cytiva, DC, US), penicillin (100 U/mL) and streptomycin (100 μg/mL), and maintained in a humidified 5% CO_2_ incubator at 37 °C. HEK293T cells were cultured in Dulbecco’s Modified Eagle Medium (DMEM) without Hepes (Lonza, Basel, Switzerland) supplemented with 10% [v/v] inactivated fetal calf serum, penicillin (100 U/mL) and streptomycin (100 μg/mL), and maintained in a humidified 10% CO_2_ incubator at 37 °C. THP-1 monocytes were obtained from ATCC. Human red blood cells were purified from blood discarded from the local blood bank.

### Virus

HIV-1 NL4-3 Ba-L was produced by transient transfection of HEK293T cells with pNL4-3 Ba-L using the calcium phosphate method. The infectious virus was harvested at 48 and 72 h after transfection and filtered through a 0.22 μm filter. NL4-3 Ba-L titers were determined on TZM-bl cells.

### Inhibition of HIV-1 replication in U87-CD4-CCR5 cells

Compounds were first dissolved in DMSO to reach a concentration of 20 mM. They were further diluted to a 200 µM working stock and then further diluted to reach a starting concentration of 10 µM in the culture plate. U87-CD4-CCR5 cells were seeded in quadruplicate at 4000 cells/well in a final volume of 100 µL. The next day, 24 µL of the compound was added in serial 1:2 dilutions to the cells. Two hours later, 25 µL of infectious virus (NL4-3 Ba-L) were added to the cells with a TCID_50_ of 250. AZT at 10 µM was included as an inhibition control. The medium was replenished in the presence of compounds once every 3 to 4 days. The supernatant was collected every 3–4 days and analyzed for virus production by an in-house p24 capture ELISA. IC_50_ was calculated using “Quest Graph™ IC_50_ Calculator” (AAT Bioquest, Inc., https://www.aatbio.com/tools/ic50-calculator) [44]. Cytotoxicity of the compound was tested on U87-CD4-CCR5 cells in parallel by MTT assay (at the same protocol as in Sect. 3.5 but after 11 days of incubation))[39].

### qPCR quantification of reverse transcribed and integrated DNA

U87-CD4-CCR5 cells were plated in a 24-wells plate at 1.25 × 10^5^ cells per well. After preincubation of the cells for 18 h with compound 696 at 10 µM, or AZT at 10 µM as an inhibition control, cells were infected with 1000 TCID_50_ of NL4-3 Ba-L (DNAse treated for 30 min). Total DNA was extracted 48 h post-infection using the AllPrep DNA/RNA Mini Kit (Qiagen, Venlo, Netherlands). The number of viral HIV-1 reverse-transcribed DNA copies in the infected cells were measured by qPCR, using GoTaq qPCR Master mix 2X (Promega, WS, US) and a primer pair detecting *pol* viral DNA: Pol E 5’ TTA ACC TGC CAC CTG TAG TAG C 3’ and Pol B: 5’ ATG TGT ACA ATC TAG TTG CC 3’. β-actin copy numbers were determined to correct for DNA input using primer pair: BA-S 5’ GGG TCA GAA GGA TTC CTA TG 3’ and BA-AS 5’ GGT CTC AAA CAT GAT CTG GG 3’. The following program was used in the LightCycler 480 Real-Time PCR System (Roche, Basel, Switzerland): pre-incubation: 95^o^ C for 3 min, pre-amplification: 2 cycles of 95 °C for 15 s and 49^o^ C for 15 s, amplification: 40 cycles of 95 °C for 10 s, 58^o^ C for 20 s, 72 °C for 30 s, melting curve stage: 95^o^ C for 5 s, 55^o^ C for 1 min and then 97^o^ C with acquisition every 10^o^ C, cooling stage: 40^o^ C for 10 s. Serial dilution of the 8E5 cell line which contains 1 copy of HIV-1 proviral DNA per cell was used as a standard curve for viral RT products [46]. This cell line was obtained through the NIH AIDS Reagent Program, Division of AIDS, NIAID, NIH [47]. Results were calculated as the number of copies of Pol per copy of β-actin. Statistical analysis was performed using an unpaired, two-tailed, Mann–Whitney test with GraphPad Prism 7.0 software. Tests were performed in quadruplicate, in two independent experiments.

### Affinity assays

The affinity of compound 696 for HIV-1 CA in vitro was measured by microscale thermophoresis (MST) as described previously for FIV CA [31]. Due to its intrinsic autofluorescence interfering with MST, the binding of compound 314 to CA was measured using isothermal microcalorimetry (ITC) with a Microcal iTC200 calorimeter (Malvern Instruments, Worcestershire, UK). The calorimeter cell contained 200 µL of recombinant HIV-1 CA (kind gifted by Drs Verrier and Coiffier [48]) at 0.78 mg/mL in phosphate buffer (NaH_2_PO_4_/Na_2_HPO_4_) 50 mM pH 7.5, NaCl 100 mM, 1% DMSO [v/v]. Compound 314 at 200 µM was then injected sequentially into the calorimeter cell (injection #1: 1 µL, injections #2-#20: 2 µL) at 120 s intervals. Data were analyzed using the OriginLab8.5® software according to the manufacturer’s instructions.

### Molecular docking

The molecular docking experiments were mostly performed as previously described [31]. The hexameric crystal structure of the HIV-1 capsid protein CA was used as the target (PDB entry 4XFX [19]). Compounds were modeled using the smiles code from the ChemOffice software. Then, CA and compounds were prepared using AutoDockTools v1.5.6 [49]. The polar hydrogen atoms were added, the non-polar hydrogens were merged, and the Gasteiger partial atomic charges were computed. Finally, all the possible rotatable bonds were assigned for each compound molecule.

A “blind docking” was then carried out with the program AutoDock Vina v1.1.2 [50]. Compounds were treated as flexible while the target protein was treated as rigid. The search space was defined to encompass the entire surface of the target protein, and a large exhaustiveness value of 500 was used regarding the wide search space (70 × 60 × 70 Å^3^). A visual examination of the resulting docking poses was carried out using PyMOL (Schrödinger, LLC).

## Conclusions

As it shows good metabolic stability and low unspecific toxicity, as it targets a region of CA which appears promising in a therapeutic goal, as it inhibits HIV-1 replication in vitro even though it has not been optimized, and given its low-cost synthesis, compound 696 represents an interesting lead molecule which warrants further optimization to generate potential anti-HIV therapeutic derivatives. Moreover, as compounds with closely related structures did not demonstrate any inhibition of viral replication, we concluded that this benzimidazole system is very specific in its antiviral effects.

## Supplementary Information

**Additional file 1. **Details of the synthesis and characterization of the compounds, and structures of all the assayed molecules.

## Data Availability

The data-sets used and/or analysed during the current study available from the corresponding author on reasonable request.

## References

[1] de Mendoza C (2019). UNAIDS Update Global HIV Numbers. AIDS Rev.

[2] Weber IT, Harrison RW (2017). Decoding HIV resistance: from genotype to therapy. Future Med Chem.

[3] Foley B, Leitner T, Apetrei C, Hahn B, Mizrachi I, Mullins J, Rambaut A, Wolinsky S, Korber B (2018). HIV Sequence Compendium 2018.

[4] Novikova M, Zhang Y, Freed EO, Peng K (2019). Multiple Roles of HIV-1 Capsid during the Virus Replication Cycle. Virol Sin.

[5] Fassati A (2012). Multiple roles of the capsid protein in the early steps of HIV-1 infection. Virus Res.

[6] Thali M, Bukovsky A, Kondo E, Rosenwirth B, Walsh CT, Sodroski J, Gottlinger HG (1994). Functional association of cyclophilin A with HIV-1 virions. Nature.

[7] Kong LB, An D, Ackerson B, Canon J, Rey O, Chen IS, Krogstad P, Stewart PL (1998). Cryoelectron microscopic examination of human immunodeficiency virus type 1 virions with mutations in the cyclophilin A binding loop. J Virol.

[8] Price AJ, Jacques DA, McEwan WA, Fletcher AJ, Essig S, Chin JW, Halambage UD, Aiken C, James LC (2014). Host cofactors and pharmacologic ligands share an essential interface in HIV-1 capsid that is lost upon disassembly. PLoS Pathog.

[9] Bejarano DA, Peng K, Laketa V, Borner K, Jost KL, Lucic B, Glass B, Lusic M, Muller B, Krausslich HG (2019). HIV-1 nuclear import in macrophages is regulated by CPSF6-capsid interactions at the nuclear pore complex. Elife.

[10] Matreyek KA, Yucel SS, Li X, Engelman A (2013). Nucleoporin NUP153 phenylalanine-glycine motifs engage a common binding pocket within the HIV-1 capsid protein to mediate lentiviral infectivity. PLoS Pathog.

[11] Sayah DM, Sokolskaja E, Berthoux L, Luban J (2004). Cyclophilin A retrotransposition into TRIM5 explains owl monkey resistance to HIV-1. Nature.

[12] Spearman P (2016). HIV-1 Gag as an Antiviral Target: Development of Assembly and Maturation Inhibitors. Curr Top Med Chem.

[13] Folio C, Sierra N, Dujardin M, Alvarez G, Guillon C (2017). Crystal structure of the full-length Feline Immunodeficiency Virus capsid protein shows an N-terminal beta-hairpin in the absence of N-terminal proline. Viruses.

[14] Jin Z, Jin L, Peterson DL, Lawson CL (1999). Model for lentivirus capsid core assembly based on crystal dimers of EIAV p26. J Mol Biol.

[15] Momany C, Kovari LC, Prongay AJ, Keller W, Gitti RK, Lee BM, Gorbalenya AE, Tong L, McClure J, Ehrlich LS, Summers MF, Carter C, Rossmann MG (1996). Crystal structure of dimeric HIV-1 capsid protein. Nat Struct Biol.

[16] Jacques DA, McEwan WA, Hilditch L, Price AJ, Towers GJ, James LC (2016). HIV-1 uses dynamic capsid pores to import nucleotides and fuel encapsidated DNA synthesis. Nature.

[17] Obal G, Trajtenberg F, Carrion F, Tome L, Larrieux N, Zhang X, Pritsch O, Buschiazzo A (2015). STRUCTURAL VIROLOGY. Conformational plasticity of a native retroviral capsid revealed by x-ray crystallography. Science.

[18] Pornillos O, Ganser-Pornillos BK, Kelly BN, Hua Y, Whitby FG, Stout CD, Sundquist WI, Hill CP, Yeager M (2009). X-ray structures of the hexameric building block of the HIV capsid. Cell.

[19] Gres AT, Kirby KA, KewalRamani VN, Tanner JJ, Pornillos O, Sarafianos SG (2015). X-Ray crystal structures of native HIV-1 capsid protein reveal conformational variability. Science.

[20] Carnes SK, Sheehan JH, Aiken C (2018). Inhibitors of the HIV-1 capsid, a target of opportunity. Curr Opin HIV AIDS.

[21] McArthur C, Gallazzi F, Quinn TP, Singh K (2019). HIV Capsid Inhibitors Beyond PF74. Diseases.

[22] Vernekar SKV, Sahani RL, Casey MC, Kankanala J, Wang L, Kirby KA, Du H, Zhang H, Tedbury PR, Xie J, Sarafianos SG, Wang Z (2020). Toward Structurally Novel and Metabolically Stable HIV-1 Capsid-Targeting Small Molecules. Viruses.

[23] Ternois F, Sticht J, Duquerroy S, Krausslich HG, Rey FA (2005). The HIV-1 capsid protein C-terminal domain in complex with a virus assembly inhibitor. Nat Struct Mol Biol.

[24] Kelly BN, Kyere S, Kinde I, Tang C, Howard BR, Robinson H, Sundquist WI, Summers MF, Hill CP (2007). Structure of the antiviral assembly inhibitor CAP-1 complex with the HIV-1 CA protein. J Mol Biol.

[25] Lemke CT, Titolo S, Goudreau N, Faucher AM, Mason SW, Bonneau P (2013). A novel inhibitor-binding site on the HIV-1 capsid N-terminal domain leads to improved crystallization via compound-mediated dimerization. Acta Crystallogr D Biol Crystallogr.

[26] Link JO, Rhee MS, Tse WC, Zheng J, Somoza JR, Rowe W, Begley R, Chiu A, Mulato A, Hansen D, Singer E, Tsai LK, Bam RA, Chou CH, Canales E, Brizgys G, Zhang JR, Li J, Graupe M, Morganelli P, Liu Q, Wu Q, Halcomb RL, Saito RD, Schroeder SD, Lazerwith SE, Bondy S, Jin D, Hung M, Novikov N, Liu X, Villasenor AG, Cannizzaro CE, Hu EY, Anderson RL, Appleby TC, Lu B, Mwangi J, Liclican A, Niedziela-Majka A, Papalia GA, Wong MH, Leavitt SA, Xu Y, Koditek D, Stepan GJ, Yu H, Pagratis N, Clancy S, Ahmadyar S, Cai TZ, Sellers S, Wolckenhauer SA, Ling J, Callebaut C, Margot N, Ram RR, Liu YP, Hyland R, Sinclair GI, Ruane PJ, Crofoot GE, McDonald CK, Brainard DM, Lad L, Swaminathan S, Sundquist WI, Sakowicz R, Chester AE, Lee WE, Daar ES, Yant SR, Cihlar T (2020). Clinical targeting of HIV capsid protein with a long-acting small molecule. Nature.

[27] Long M, Cantrelle FX, Robert X, Boll E, Sierra N, Gouet P, Hanoulle X, Alvarez GI, Guillon C (2021). Identification of a Potential Inhibitor of the FIV p24 Capsid Protein and Characterization of Its Binding Site. Biochemistry.

[28] Ríos N, Chavarría C, Gil C, Porcal W (2013). Microwave-Assisted Solid-Phase Synthesis of a 1, 2-Disubstituted Benzimidazole Library by Using a Phosphonium Linker. J Heterocycl Chem.

[29] Alvarez G, Varela J, Cruces E, Fernandez M, Gabay M, Leal SM, Escobar P, Sanabria L, Serna E, Torres S, Figueredo Thiel SJ, Yaluff G, Vera de Bilbao NI, Cerecetto H, Gonzalez M (2015). Identification of a new amide-containing thiazole as a drug candidate for treatment of Chagas’ disease. Antimicrob Agents Chemother.

[30] Sierra N, Folio C, Robert X, Long M, Guillon C, Alvarez G (2018). Looking for novel capsid protein multimerization inhibitors of Feline Immunodeficiency Virus. Pharmaceuticals.

[31] Xu JP, Francis AC, Meuser ME, Cocklin S (2018). Exploring Modifications of an HIV-1 Capsid Inhibitor: Design, Synthesis, and Mechanism of Action. J Drug Des Res.

[32] Alvarez G, Varela J, Marquez P, Gabay M, Arias Rivas CE, Cuchilla K, Echeverria GA, Piro OE, Chorilli M, Leal SM, Escobar P, Serna E, Torres S, Yaluff G, Vera de Bilbao NI, Gonzalez M, Cerecetto H (2014). Optimization of antitrypanosomatid agents: identification of nonmutagenic drug candidates with in vivo activity. J Med Chem.

[33] Aguilera E, Varela J, Birriel E, Serna E, Torres S, Yaluff G, de Bilbao NV, Aguirre-Lopez B, Cabrera N, Diaz Mazariegos S, de Gomez-Puyou MT, Gomez-Puyou A, Perez-Montfort R, Minini L, Merlino A, Cerecetto H, Gonzalez M, Alvarez G (2016). Potent and Selective Inhibitors of Trypanosoma cruzi Triosephosphate Isomerase with Concomitant Inhibition of Cruzipain: Inhibition of Parasite Growth through Multitarget Activity. ChemMedChem.

[34] Burdick RC, Li C, Munshi M, Rawson JMO, Nagashima K, Hu WS, Pathak VK (2020). HIV-1 uncoats in the nucleus near sites of integration. Proc Natl Acad Sci U S A.

[35] Krissinel E, Henrick K (2007). Inference of macromolecular assemblies from crystalline state. J Mol Biol.

[36] Balasubramaniam M, Zhou J, Addai A, Martinez P, Pandhare J, Aiken C, Dash C (2019). PF74 Inhibits HIV-1 Integration by Altering the Composition of the Preintegration Complex. J Virol.

[37] Taylor RD, MacCoss M, Lawson AD (2014). Rings in drugs. J Med Chem.

[38] Alvarez G, Martinez J, Varela J, Birriel E, Cruces E, Gabay M, Leal SM, Escobar P, Aguirre-Lopez B, Cabrera N, Tuena de Gomez-Puyou M, Gomez Puyou A, Perez-Montfort R, Yaluff G, Torres S, Serna E, Vera de Bilbao N, Gonzalez M, Cerecetto H (2015). Development of bis-thiazoles as inhibitors of triosephosphate isomerase from Trypanosoma cruzi. Identification of new non-mutagenic agents that are active in vivo. Eur J Med Chem.

[39] Aguilera E, Perdomo C, Espindola A, Corvo I, Faral-Tello P, Robello C, Serna E, Benítez F, Riveros R, Torres S, Vera de Bilbao NI, Yaluff G, Alvarez G (2019). A nature-inspired design yields a new class of steroids against trypanosomatids. Molecules.

[40] Boiani M, Merlino A, Gerpe A, Porcal W, Croce F, Depaula S, Rodriguez MA, Cerecetto H, Gonzalez M (2009). o-Nitroanilines as major metabolic products of anti-Trypanosoma cruzi 5-phenylethenylbenzofuroxans in microsomal and cytosolic fractions of rat hepatocytes and in whole parasitic cells. Xenobiotica.

[41] Sushko I, Novotarskyi S, Korner R, Pandey AK, Cherkasov A, Li J, Gramatica P, Hansen K, Schroeter T, Muller KR, Xi L, Liu H, Yao X, Oberg T, Hormozdiari F, Dao P, Sahinalp C, Todeschini R, Polishchuk P, Artemenko A, Kuzmin V, Martin TM, Young DM, Fourches D, Muratov E, Tropsha A, Baskin I, Horvath D, Marcou G, Muller C, Varnek A, Prokopenko VV, Tetko IV (2010). Applicability domains for classification problems: Benchmarking of distance to models for Ames mutagenicity set. J Chem Inf Model.

[42] Matiadis D, Saporiti T, Aguilera E, Robert X, Guillon C, Cabrera N, Perez-Montfort R, Sagnou M, Alvarez G (2021). Pyrazol(in)e derivatives of curcumin analogs as a new class of anti-Trypanosoma cruzi agents. Future Med Chem.

[43] Serrière J, Fenel D, Schoehn G, Gouet P, Guillon C (2013). Biophysical characterization of the Feline Immunodeficiency Virus p24 Capsid protein conformation and in vitro capsid assembly. PLoS ONE.

[44] Bjorndal A, Deng H, Jansson M, Fiore JR, Colognesi C, Karlsson A, Albert J, Scarlatti G, Littman DR, Fenyo EM (1997). Coreceptor usage of primary human immunodeficiency virus type 1 isolates varies according to biological phenotype. J Virol.

[45] Platt EJ, Wehrly K, Kuhmann SE, Chesebro B, Kabat D (1998). Effects of CCR5 and CD4 cell surface concentrations on infections by macrophagetropic isolates of human immunodeficiency virus type 1. J Virol.

[46] Kootstra NA, Schuitemaker H (1999). Phenotype of HIV-1 lacking a functional nuclear localization signal in matrix protein of gag and Vpr is comparable to wild-type HIV-1 in primary macrophages. Virology.

[47] Folks TM, Powell D, Lightfoote M, Koenig S, Fauci AS, Benn S, Rabson A, Daugherty D, Gendelman HE, Hoggan MD (1986). Biological and biochemical characterization of a cloned Leu-3- cell surviving infection with the acquired immune deficiency syndrome retrovirus. J Exp Med.

[48] Guillon C, Mayol K, Terrat C, Compagnon C, Primard C, Charles M-H, Delair T, Munier S, Verrier B (2007). Formulation of HIV-1 Tat and p24 antigens by PLA nanoparticles or MF59 impacts the breadth, but not the magnitude, of serum and faecal antibody responses in rabbits. Vaccine.

[49] Morris GM, Huey R, Lindstrom W, Sanner MF, Belew RK, Goodsell DS, Olson AJ (2009). AutoDock4 and AutoDockTools4: Automated docking with selective receptor flexibility. J Comput Chem.

[50] Trott O, Olson AJ (2010). AutoDock Vina: improving the speed and accuracy of docking with a new scoring function, efficient optimization, and multithreading. J Comput Chem.

