# Supplementary material for: Identification of 2-(4-N,N-Dimethylaminophenyl)-5-methyl-1-phenethyl-1H-benzimidazole targeting HIV-1 CA capsid protein and inhibiting HIV-1 replication in cellulo
Source: BMC Pharmacol Toxicol. 2022 Jun 28;23:43. doi: 10.1186/s40360-022-00581-7 (PMC9241302; doi:10.1186/s40360-022-00581-7)
Supplement: Supplementary file 1 — Additional file 1. Details of the synthesis and characterization of the compounds, and structures of all the assayed molecules. [file 40360_2022_581_MOESM1_ESM.docx]

Identification of 2-(4-N,N-Dimethylaminophenyl)-5-methyl-1-phenethyl-1H-benzimidazole targeting HIV-1 CA capsid protein and inhibiting HIV-1 replication *in cellulo*

Guzmán Alvarez ^a‡*^, Lisa van Pul ^b‡^, Xavier Robert ^c^, Zoraima Artía ^a^, Ad C. van Nuenen ^b^, Mathieu Long ^c^, Natalia Sierra ^a^, Williams Porcal ^d^, Neeltje A. Kootstra ^b*^, Christophe Guillon ^c*^.

a- Laboratorio de Moléculas Bioactivas, Departamento de Ciencias Biológicas, CENUR Litoral Norte, Universidad de la República, 60000 Paysandú, Uruguay.

b- Department of Experimental Immunology, Amsterdam UMC, Amsterdam Infection & Immunity Institute, University of Amsterdam, 1105 AZ Amsterdam, the Netherlands.

c- Retroviruses and Structural Biochemistry, UMR5086, Université de Lyon, CNRS, MMSB, 69367 Lyon, France.

d- Departamento de Química Orgánica, Facultad de Química, Universidad de la República, 11800 Montevideo, Uruguay.

Preparation of the compound **696**

**Scheme S1**. Synthetic steps for the preparation of **696** ^1^.

Step 1. 2-fluoro-4-methyl-1-nitrobenzene (1.3 g, 7.02 mmol, 1.0 equiv.) phenylethylamine (849.4 mg, 7.02 mmol, 1.0 equiv.) was dissolved in 10 mL anhydrous *N, N*-Dimethylformamide, then K_2_CO_3_ (2421 mg, 17.55 mmol) was added to the solution. The resultant was stirred at 40 ºC in oil bath for 16 hours. Subsequently, the mixture was extracted with ethyl acetate (30 mL) for one time, the aqueous phase was isolated and aqueous hydrochloric acid solution (1 M) was added into the solution to adjust the pH value to 3-4, Next, the mixture was extracted with ethyl acetate (30 mL x3) in three times. The organic phase was combined and dried by Na_2_SO_4_. The product **A** was obtained by flash column chromatography on silica gel using dichloromethane/methanol (20:1, v/v) as eluent (1.698 g, yield: 80%).

Step 2. 5-methyl-2-nitro-N-phenethylaniline (1698 mg, 5.94 mmol, 1.0 equiv.), Zinc powder (971 mg, 2.5 equiv., activated by diluted hydrochloric acid) and NH_4_Cl (1588 mg, 5 equiv.) were added in tetrahydrofuran (30 mL) and reflux under N_2_ atmosphere, and monitored by TLC (Thin Layer Chromatography) until completion. After negative-pressure filtration, the organic solvent was evaporated, the resulting residue was purified by column chromatography on silica gel using dichloromethane/methanol (50:1, v/v) as eluent to give compound **B** (960 mg, yield: 60%).

Step 3. An equimolar (1.0 mmol) mixture of 5-methyl-N1-phenethylbenzene-1,2-diamine, aromatic aldehyde (4-(dimethylamino)benzaldehyde), and L-Carnitine (10 mol %) was vigorously stirred at 60°C in ethanol (3 mL) for the specific time indicated by TLC (petroleum: ethyl acetate ether = 4:1). After completion of the reaction, the mixture was quenched by adding water (20 mL), extracted with ethyl acetate (3 x 10 mL), and the combined extracts were dried by anhydrous MgSO_4_. The filtrate was evaporated and the **696** was obtained as the only product.

**2-(4-N,N-Dimethylaminophenyl)-5-methyl-1-phenethyl-1H- benzimidazole (696)**. Yellow solid; yield: 70 %, mp 120–121, Purity >98%, ºC ^1^HNMR (CDCl_3_) d (ppm): 2.52 (s, 3H), 3.06 (s, 6H), 3.12 (t, J= 8.0Hz,2H),4.44(t, J=8.0Hz, 2H), 6.75 (d, J= 9.2Hz,2H),7.05(m, 2H), 7.25 (m,3H),7.13 (dd, J= 0.8 Hz, J=8.0Hz, 1H), 7.29 (d, J= 8.4Hz,1H),7.47 (d, J= 8.8 Hz, 2H), 7.65 (s, 1H). 13C NMR (CDCl_3_): d 22.1, 36.1, 40.3, 46.7, 109.3, 112.3, 118.5, 123.2, 120.2, 124.2, 128.2, 129.5, 130.4, 134.0, 137.1, 154.3. EI–MS, m/z (abundance, %): 355 (M+, 100), 264 (93), 220 (17), 145 (13). Anal. Calc. for C_24_H_25_N_3_: C, 81.09; H, 7.09; N, 11.82. Found: C, 81.36; H, 7.42; N, 11.67.

Preparation of **314**

**Scheme S2**. Synthetic steps for the preparation of **314**

Step 1. A mixture of the corresponding aldehyde (1.05 equiv.), the corresponding thiosemicarbazide (1.00 equiv.), catalytic amount of p-toluenesulfonic acid, and dry toluene (1 mL per 100 mg of aldehyde) was stirred at room temperature until disappearance of the aldehyde (12−24 h, checked by TLC, SiO_2_, using as a mobile phase petroleum ether/ethyl acetate 70:30). After that, the precipitate was filtered off and washed with petroleum ether. The solid was crystallized from ethanol.

Step 2. A mixture of the corresponding thiosemicarbazide (1.0 equiv.), bromopyruvic acid (1.2 equiv.), and ethanol 98% (1 mL per 100 mg of thiosemicarbazide) was heated at reflux until disappearance of the thiosemicarbazide (4− 10 h, checked by TLC, SiO_2_, and petroleum ether/ ethyl acetate 70:30). After that, the mixture was cooled at room temperature, and the precipitate was filtered off and washed with ethanol/water (80:20). The solid was crystallized from ethanol or ethanol/water.

Step 3. A mixture of the corresponding acid (1.0 equiv.), thionyl chloride (1.2 equiv.) and dry toluene (1 mL per 100 mg of acid) was heated at 100 ^o^C for 1 h. After that, a solution of the corresponding amine (1.0 equiv.) and triethylamine (5.0 equiv.) were added dropwise during 30 min to the reaction cooled at 0 ^o^C. The mixture of reaction was stirred at room temperature until disappearance of the activated acid (12-24 h, checked by TLC, Al_2_O_3_, petroleum ether: ethyl acetate 70:30). After that, the solvent was evaporated *in vacuo* and the residue was partitioned between methylene dichloride and saturated aqueous solution of sodium bicarbonate. The organic layer was washed with aqueous phosphate buffer (pH 4-5), dried with anhydrous sodium sulfate and evaporated *in vacuo*. The desired product was purified, from the residue of evaporation, by column chromatography (Al_2_O_3_, petroleum ether: ethyl acetate 0 to 40 %).

**(*2E,2Z*)-3-Allyl-4-[((*E*)-4-cinnamylpiperazin-1-yl)carbonyl]-2-[2-((*E*)-3-(furan-2- yl)propenylidene)hydrazono]-2,3-dihydrothiazole (314).** Yellow solid, yield: 60 %, mp 157-159 ^o^C, Purity >98%, ºC ^1^HNMR (CDCl_3_) d (ppm): 2.52 (bs, 4H), 3.2 (d, J= 6.6 Hz, 2H), 3.68 (bs, 4H), 4.66 (d, J= 5.6 Hz, 2H), 5.19 (m, 2H), 5.91 (m, 1H), 6.15 (s,1H), 6.25 (m, 1H), 6.46 (m, 1H), 6.56 d, J=16Hz, 1H), 6.64 (d, J=16, 1H), 6.92 (dd, J=9.9/16, 1H), 7.38 (m, 4H), 7.44 (m, 1H), 8.07 (d, J=9.9, 1H). 13C NMR (CDCl_3_): d 142, 110,132, 125, 152, 168, 104, 142, 170, 42, 47, 60, 124, 134, 128, 118. MS (EI) *m/z* (abundance, %): 487 (M+., 13), 407 (10), 117 (100). UV: 387 nm (ε = 27.2 ± 0.4 cm^-1^mM^-1^). Found: C, 66.6; H, 6.0; N, 14.1; S, 6.4. C_27_H_29_N_5_O_2_S m/e: 487.20 (100.0%), 488.21 (29.6%), 489.20 (5.1%), 489.21 (4.9%), 488.20 (2.6%), 490.20 (1.4%) C, 66.50; H, 5.99; N, 14.36; O, 6.56; S, 6.58.

**Experimental Characterization of compounds.**

All of the synthesized compounds were chemically characterized by thin layer chromatography (TLC), nuclear magnetic resonance (1H NMR, 13C NMR), and elemental microanalyses (CHN). Alugram SIL G/UV254 (Layer: 0.2 mm) (Macherey-Nagel GmbH & Co. KG., Düren, Germany) was used for TLC, and silica gel 60 (0.040−0.063 mm, Merck) was used for flash column chromatography. The NMR spectra were recorded on a Bruker DPX 400 (400 MHz for 1H and 100 MHz for 13C), using TMS as the internal standard and with the indicated deuterated solvent; the chemical shifts are reported in ppm (δ) and coupling constants (J) values are given in Hertz (Hz). Signal multiplicities are represented by s (singlet), d (doublet), dd (double

doblet), t (triplet), tt (triple triplet), and m (multiplet). Structural assignments were corroborated by HMBC and HSQC experiments. Mass spectrometry experiments were performed on a HEWLETT PACKARD MSD 5973 or a LC/MSD-Serie 100 using electronic impact (EI) or electrospray ionization (ESI), respectively. To determine the purity of the compounds, elemental microanalyses obtained on a Carlo Erba Model EA1108 elemental analyzer from vacuum-dried samples were used. The analytical results for C, H, and N were within ±0.4 of the theoretical values. Melting points were recorded on ELECTROTHERMAL IA-9100 equipment, and they were not corrected. Samples of all product of this work are available from the authors.

**Theoretical analysis of Compounds.**

The active compounds (**314** and **696**) were tested online following the recommendations. These were tested by four different on-line softwares for PAINS and aggregator compounds. (<http://zinc15.docking.org/patterns/home>, <http://fafdrugs3.mti.univparis-diderot.fr>, /http://www.cbligand.org/PAINS/, http://advisor.docking.org). None of them show problem in those tests (fig S1 for compound 696 and data not shown). Moreover, compounds 314 and 696 had already been used in screening assays against many other targets and were not inhibitor ^3-6^.

Figure S1: on-line characterization of compound 696 for PAINS and aggregator compounds

**Table S1.** Predicted pharmacokinetic parameters and toxicology of compounds 69, 314 and reference compound PF74.

| **Compound** | **Solubility (mg/mL)** | **Gastrointestinal**  **Absorption** | **Brain Permeability** | **CYP**  **inhibitor** | **P-gp***  **substrate** | **LogP** | PAINS [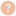](http://www.swissadme.ch/index.php) | **Mutagenicity**** | **Oral Rat **LD50 mg/kg** |
| --- | --- | --- | --- | --- | --- | --- | --- | --- | --- |
| **696** | 6.44e-04 | HIGH | YES | YES | YES | 4.8 | No alerts | NO** | 2820** |
| **314** | 3.45e-03 | HIGH | NO | YES | NO | 4.2 | 1 alert: imine_imine_B [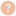](http://www.swissadme.ch/index.php) | NO | >2000 |
| **PF74** | 1.87e-03 | HIGH | YES | YES | YES | 4.1 | 1 alert: indol_3yl_alk | NO** | 1167** |

*P-glycoprotein substrate, **Calculated by Toxicity Estimation Software Tool (TEST)

| Table S2. List of all compounds tested in cellulo. | | | |
| --- | --- | --- | --- |
| Internal code |  | Internal code |  |
| ID696 |  | Nr-0922c |  |
| Nr-0937 |  | G5 |  |
| Nr-1068a |  | G6 |  |
| G7 |  | G8 |  |
| G9 |  | G10 |  |
| G11 |  | G16 |  |
| Nr-11130 |  | Nr-12134 |  |
| Nr-12136 |  | G15 |  |
| ID878 |  | ID1310 |  |
| ID553 |  | ID314 |  |
| G17 |  | ID305 |  |

Figure S2: Thin Layer Chromatography of 696 after incubation 4h in microsomal fraction (MF) and cytosolic fraction (CF).

**
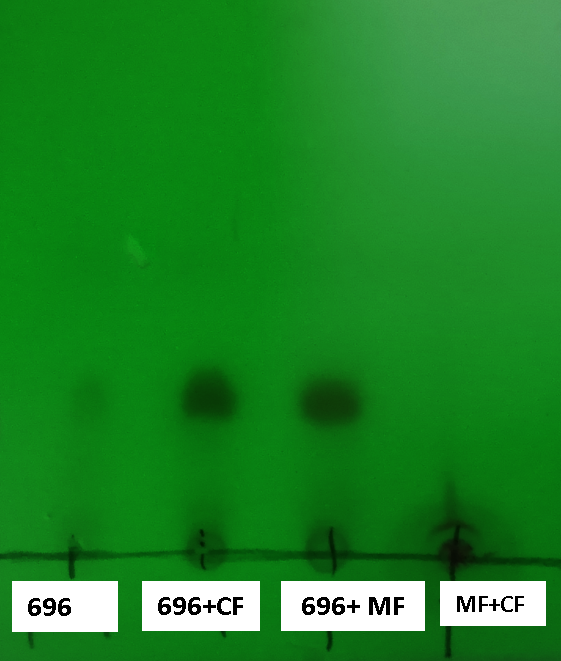
**

**Supplementary references**

1- Long, M.; Cantrelle, F. X.; Robert, X.; Boll, E.; Sierra, N.; Gouet, P.; Hanoulle, X.; Alvarez, G. I.; Guillon, C., 2021. Identification of a Potential Inhibitor of the FIV p24 Capsid Protein and Characterization of Its Binding Site. *Biochemistry*, in press.

2 - Aldrich C, Bertozzi C, Georg GI, Kiessling L, Lindsley C, Liotta D, Merz KM Jr, Schepartz A, Wang S., 2017. The Ecstasy and Agony of Assay Interference Compounds. *ACS Med Chem Lett.* 8, 379-382.

3- Risi, G., Aguilera, E., Ladós, E., Suárez, G., Carrera, I., Álvarez, G., Salinas, G., 2019. Caenorhabditis elegans Infrared-Based Motility Assay Identified New Hits for Nematicide Drug Development. *Vet. Sci.* 6, 29.

4- Saramago, L., Gomes, H., Aguilera, E., Cerecetto, H., González, M., Cabrera, M., Alzugaray, M., da Silva Vaz Junior, I., Nunes da Fonseca, R., Aguirre-López, B., Cabrera, N., Pérez-Montfort, R., Merlino, A., Moraes, J., Álvarez, G., 2018. Novel and Selective Rhipicephalus microplus Triosephosphate Isomerase Inhibitors with Acaricidal Activity. *Vet. Sci.* 5, 74.

5- Ferraro, F., Corvo, I., Bergalli, L., Ilarraz, A., Cabrera, M., Gil, J., Susuki, B.M., Caffrey, C.R., Timson, D.J., Robert, X., Guillon, C., Freire, T., Álvarez, G., 2020. Novel and selective inactivators of Triosephosphate isomerase with anti-trematode activity. *Sci. Rep.* 10, 1–13.

6- Aguilera, E., Varela, J., Birriel, E., Serna, E., Torres, S., Yaluff, G., de Bilbao, N.V.N.V., Aguirre-López, B., Cabrera, N., Díaz Mazariegos, S., de Gómez-Puyou, M.T.M.T., Gómez-Puyou, A., Pérez-Montfort, R., Minini, L., Merlino, A., Cerecetto, H., González, M., Alvarez, G., 2016. Potent and Selective Inhibitors of Trypanosoma cruzi Triosephosphate Isomerase with Concomitant Inhibition of Cruzipain: Inhibition of Parasite Growth through Multitarget Activity. *ChemMedChem* 11, 1328-1338.
